# Septins in Infections: Focus on Viruses

**DOI:** 10.3390/pathogens10030278

**Published:** 2021-03-02

**Authors:** Thomas Henzi, Nils Lannes, Luis Filgueira

**Affiliations:** Anatomy Unit, Department of Oncology, Microbiology and Immunology, Faculty of Science and Medicine, University of Fribourg, 1700 Fribourg, Switzerland; thomas.henzi@unifr.ch (T.H.); nils.lannes@unifr.ch (N.L.)

**Keywords:** virus infection, cytoskeleton, septin

## Abstract

Human septins comprise a family of 13 genes that encode conserved GTP-binding proteins. They form nonpolar complexes, which assemble into higher-order structures, such as bundles, scaffolding structures, or rings. Septins are counted among the cytoskeletal elements. They interact with the actin and microtubule networks and can bind to membranes. Many cellular functions with septin participation have been described in the literature, including cytokinesis, motility, forming of scaffolding platforms or lateral diffusion barriers, vesicle transport, exocytosis, and recognition of micron-scale curvature. Septin dysfunction has been implicated in diverse human pathologies, including neurodegeneration and tumorigenesis. Moreover, septins are thought to affect the outcome of host–microbe interactions. Implication of septins has been demonstrated in fungal, bacterial, and viral infections. Knowledge on the precise function of a particular septin in the different steps of the virus infection and replication cycle is still limited. Published data for vaccinia virus (VACV), hepatitis C virus (HCV), influenza A virus (H1N1 and H5N1), human herpesvirus 8 (HHV-8), and Zika virus (ZIKV), all of major concern for public health, will be discussed here.

## 1. Cell Biology of Septins

Septins are a family of conserved GTPases belonging to the GTPase superclass of P-loop NTPases [1]. First described in 1971 by Hartwell [2], they have since been identified in all eukaryotes, but not in higher plants. The human genome encodes 13 septin genes classified into four homology groups based on sequence similarity (SEPT2, SEPT3, SEPT6, and SEPT7). The SETP2 group comprises SEPTIN1, SEPTIN2, SEPTIN4, and SEPTIN5; the SEPT3 group SEPTIN3, SEPTIN9, and SEPTIN12; the SEPT6 group SEPTIN6, SEPTIN8, SEPTIN10, SEPTIN11, and SEPTIN14, while SEPTIN7 is the only member of the SEPT7 group [3]. The diversity is even higher due to the existence of transcripts, expressed from alternative promoters and splicing variants [4]. Some septin family members are expressed ubiquitously (e.g., septin 2, 7, and 9), others (septin 1, 3, 12, and 14) show tissue-specific expression patterns [5].

**Figure 1:**
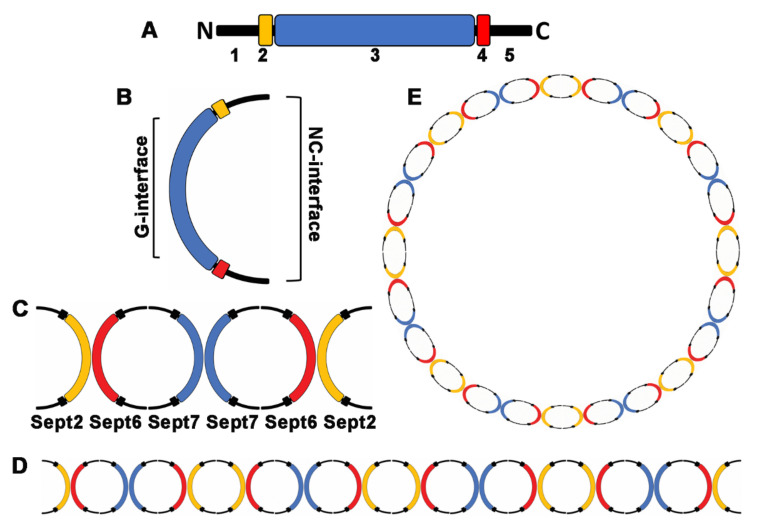
Septin domain structure and filament assembly, (**A**) septin domains 1: variable N-terminus 2: polybasic region (membrane binding) 3: GTP-binding domain 4: septin unique element (SUE) 5: variable C-terminus with coiled-coil domains (except SEPT3 group), (**B**) G- and NC-interfaces (interaction with other septins), (**C**) basic septin hexamer, (**D**) septin filament consisting of hexamers, and (**E**) septin ring.

All septins contain a highly conserved central GTP-binding domain. Many show GTPase activity, but the role of nucleotide hydrolysis is not well understood [6,7]. A short polybasic region (PBR) between the N-terminus and the GTP binding domain, able to bind to negatively charged phospholipids like phosphatidylinositol 4,5-bisphosphate (PIP2), is responsible for interactions with membranes. The septin-unique element (SUE) is located near the C-terminus. It distinguishes septins from other small GTP-binding proteins and is presumably involved in septin filament formation [8,9]. The GTP-binding domain is flanked by N- and C-terminal regions of variable length and sequence. It is assumed that the sequences of the C-terminal domains form coiled coils (with the exception of the SEPT3 group) and participate in septin–protein interactions (Figure 1).

Septin subunits interact with septin subunits from other groups via their G- and NC-interfaces. Septins assemble as heterooligomeric complexes. A basic hexameric unit consisting of septins in the order of SEPT7-SEPT6-SEPT2-SEPT2-SEPT6-SEPT7 was initially described by Sirajuddin et al. [10]. Newer findings indicate that the correct assembly of the hexamer is SEPT2–SEPT6–SEPT7–SEPT7–SEPT6–SEPT2 [11]. Addition of a member of the SEPT3 group gives rise to hetero-octameric complexes. Any given septin in a complex can be substituted by another from the same group [12]. Septin complexes polymerize end-to-end to form apolar filaments, which can associate to form higher-order structures, such as bundles, scaffolding structures, or rings. Because of their filamentous appearance and their association with cellular membranes, actin filaments and microtubules, septins are considered as the fourth component of the cytoskeleton [3].

Septins are involved in numerous biological processes including cytokinesis and motility. They serve as a regulatory module for the site-specific regulation and organization of actin and microtubule networks [13] and act as scaffolding platforms for protein recruitment to specific sites in a cell. Interactome studies for septins in general [14,15,16], as well as for individual septins [17,18] have been described. Septins may function as lateral diffusion barriers in different mammalian cell processes like cilia, flagella, dendritic spines, and yeast buds. Higher-order septin ring-like structures, tightly associated with the plasma membrane, are located at the base of these processes. Lateral diffusion of membrane associated molecules is impeded leading to subcellular compartmentalization [19]. At the base of these cellular protrusions (e.g., cilia, dendritic spines, and cytokinetic furrow), the cell membrane displays micrometer-scale curvature. Published observations indicate that recognition of micron-scale curvature is an intrinsic property of the septin cytoskeleton enabling the cell to sense its local morphology [20]. Septins take part in the intracellular transportation of vesicles. In addition, they play an active role in exocytosis. By interacting with key components of the membrane fusion machinery, they guide the docking of vesicles and their fusion with the cell membrane [18,21,22].

Septins are associated with the development of a wide variety of diseases. Knockout of the ubiquitous septin 7, septin 9, and septin 11 results in embryonic lethality [23,24]. Septin mutations and deletions are associated with male infertility [25,26], as well as neuromuscular [27] and bleeding disorders [28]. Changes in septin expression have been discovered in many cancers [19,29] and neurodevelopmental disorders (e.g., Down syndrome [30], schizophrenia [31], and bipolar disorder [32]). Histological analysis of brains of Alzheimer’s and Parkinson’s patients illustrated the presence of septins in neurofibrillary tangles and Lewy bodies [5].

The septin cytoskeleton is an essential part of the cell-autonomous immunity. To fight microbial infections and for the elimination of invasive pathogens, cells are equipped with defense mechanisms, including antimicrobial proteins, specialized degradative compartmentalization, and programmed host cell death. Cellular and animal models demonstrated that septins can sense pathogenic microbes and launch host defense mechanisms for their elimination [33]. Phagocytosis can serve as an entry mechanism for microorganisms to promote infection or be used by the host cell defense to eliminate microbes [19]. Experimental data suggest that septins contribute to phagocytic processes. Phagocytic cells express several septins, e.g., septins 2, 6, 10, and 11. Silencing of SEPT2 or SEPT11 by RNAi technology inhibited antibody-mediated phagocytosis by 50–70% [34]. The recruitment of filamentous septins may be a prerequisite for the anchorage of multiprotein complexes to form phagosomes.

To date, knowledge of septin inhibitors is still very limited. Forchlorfenuron (FCF) is the only well documented small molecule disturbing higher-order assembly of septins. FCF is a synthetic phytohormone with cytokinin activity used as a plant growth regulator. Treatment with FCF causes defects in cytokinesis and deformation of septin filaments in budding yeast [35]. It has been shown that cell proliferation is impaired and cell migration and invasion is blocked [36,37]. By interacting with the nucleotide-binding pocket of septins, FCF interferes with the GTP-binding dynamics and turnover leading to changes in polymerization and stability of septin filaments [38]. However, actin or tubulin polymerization is not affected [39]. The low toxicity level makes FCF a potential candidate for therapeutic applications of septin dependent disorders. However, off-target effects were reported before [40,41]. Modification of the chemical structure of FCF might lead to the development of new analogues with improved therapeutic potential and less side effects [42,43].

## 2. The Role of Septins in Infections

During infection, pathogens manipulate host cell components to facilitate invasion, promote replication and enable dissemination. Rearrangements of the host cell cytoskeleton are a common strategy. Likewise, the septin cytoskeleton, which in addition to actin, microtubules, and intermediate filaments is considered as the fourth component of the cytoskeleton, may also be affected. Implication of septins in microbial infections has been described for pathogenic fungi, bacteria, and viruses [8].

First, we cover here a fungal infection in plants, where fungal septins are essential for infection. The infection process of the rice blast fungus *M. oryzae* starts with the attachment to the plant surface. An appressorium develops subsequently from which a penetration peg grows out to perforate the leaf cuticle [44]. Septins colocalize with actin fibers as ring-like structures at the pore of the appressorium. Additional septin structures were found in hyphae and during invasive growth. The septin ring has a scaffolding function for F-actin, but moreover serves as a diffusion barrier to guide the alignment of specific proteins required for the buildup of a rigid penetration peg to break into the plant cell. Interestingly, fungus with septin null mutations were nonpathogenic [45].

Septins play a dual role in bacterial infections. Accumulation at the bacterial entry site and regulation of the morphology of the host cell surface promote efficient invasion [46,47,48]. On the other hand, they may participate in the cellular defense against intracellular bacterial infections, e.g., against *Shigella*. After certain bacteria, including *Shigella*, *Listeria,* and *Salmonella*, invade epithelial cells, replication may take place in the cytoplasm or within vacuoles [49]. In addition, actin tails are formed at the bacterial surface, enabling bacteria to move in the cytoplasm, avoid defense mechanisms of the host cell and infect adjacent cells [50]. However, the epithelial cell may activate defense mechanisms by inducing reorganization of septins into cage-like structures around the intracellular bacteria. Enclosed in septin cages, bacteria lose the actin-based motility and are prone to degradation by autophagy. TNF-α has been shown to enhance caging of cytosolic bacteria and to limit cell-to-cell spreading [51]. Mitochondria also contribute to efficient assembly of septin cages. Interestingly, septin cages are not formed around dead cytosolic bacteria. Thus, septins have the ability to detect live bacteria and interfere with their proliferation and spreading [52]. The combined recruitment of septins and the autophagy machinery has a stronger inhibitory effect on bacterial cell division than recruiting septins or the autophagy machinery alone [53]. Thus, septins are required to block proliferation of *Shigella*, but additional activation of the autophagy machinery is needed for high efficiency of the mechanism.

## 3. The Role of Septins in Virus Infections

The following section will present published research about the role of septins in virus infections.

### 3.1. Vaccinia Virus Infection

Firstly, the role of septins in the release of vaccinia virus (VACV) from infected cells has been reported by Pfanzelter et al. [54]. In VACV, newly assembled intracellular enveloped virus particles (IEV) are transported along microtubules to the plasma membrane [55,56]. After fusion of IEVs with the plasma membrane, two modes of viral spread are possible [57]. Either they are released from the cell as extracellular enveloped virus (EEV) promoting the long-range spread of VACV, or they remain attached to the cell membrane as so-called cell-associated enveloped viruses (CEVs). CEVs are then capable of signaling back into the cell to induce the polymerization of actin tails, facilitating spreading into and infecting adjacent cells. Endogenous septin 7 colocalizes with extracellular CEVs associated with the cell membrane, i.e., septins are recruited to viral particles after their fusion with the plasma membrane. Confocal images demonstrated that septins wrap around CEVs forming cage-like structures [54]. Actin tail formation takes place after recruitment of septins to CEVs. Upon starting of the actin polymerization, septins rapidly disappear from the viral particles. Interestingly, RNAi-mediated downregulation of septin 7 increases viral release and cell-to-cell spread of VACV. Loss of septin 2 or septin 11 produces the same effects. Thus, absence of septin 7 (or septin 2/9/11) augments the number of CEVs exhibiting an actin tail. Of note, VACV infection does not change the expression level of septin [54]. Cells infected with A36-YdF virus, a vaccinia strain unable to form actin tails, show a strongly increased number of CEVs associated with septin 7. Actin tail formation of the control strain seems to inhibit septin recruitment and/or promote its displacement from the virus. However, Arp2/3-mediated actin tail formation is not causing septin displacement from the virus [54]. Dynamin, together with formin-mediated actin polymerization, drives the separation of septins from CEVs. Similar to other viruses, VACV uses the host cell metabolism and molecular machinery for replication. To gain insight in the complex interactions with host proteins, a functional high throughput small interfering RNA screen was performed [58]. A total of 153 pro- and 149 anti-viral host factors with a strong influence on replication and viral spread were detected. Among the anti-viral factors, 2 septin proteins were found: i.e., septin 1 and septin 9. Septin 2, septin 7, and septin 11 also inhibited viral replication albeit to a minor degree. In a previous VACV siRNA screen, septin 11 has already been described as an anti-viral protein [59]. In the same study, septin 3 and septin 10 were found to have pro-viral properties.

### 3.2. Hepatitis C Virus (HCV) Infection

In hepatitis C virus (HCV) infection, Kim et al. [60] identified septin 6 to play a role. They were looking for proteins interacting with NS5b, a nonstructural HCV protein functioning as RNA-dependent RNA polymerase. They identified hnRNP A1 and septin 6 as candidates that interact with NS5b. hnRNP is an RNA-binding protein involved in pre-mRNA splicing and transport of cellular RNAs [61]. Septin 6 contributes to actin dynamics, cell shape, and microtubule regulation [3]. Both cellular proteins, hnRNP A1 and septin 6, interact with NS5b. Furthermore, hnRNP A1 and septin 6 interact also directly suggesting that the three proteins might form a replication complex. Knockdown of either septin 6 or hnRNP A1 inhibited HCV replication. Septin 6 may act as a scaffolding molecule in the replication complex connecting NS5b and hnRNP A1 via protein–protein interactions and hnRNP A1 and viral RNA via RNA–protein interaction. In this case, normal function of host hnRNP A1 and septin 6 is disrupted by HCV infection for the benefit of HCV replication. Chronic HCV infection often provokes excessive accumulation of lipid droplets (LD) causing liver steatosis, a risk factor for cirrhosis and hepatocellular carcinoma. Akil et al. [62] conducted a transcriptome analysis in human cirrhosis to investigate differential expression of septins. In HCV-induced cirrhosis samples, most septins were affected compared to normal liver samples. Septins 2, 4, 6, 7, 8, 9, and 11 were significantly upregulated, while septin 10 was unchanged. Infecting Huh7.5 cells with HCV JFH-1 (Japanese fulminate hepatitis 1) increased expression levels of septin 9. On confocal images, bundles of septin 9 containing filaments surrounding the clusters of HCV core protein were visible. The authors concluded that HCV-regulated septin 9 assembly and expression is essential for the accumulation of viral core protein. Moreover, HCV JFH-1 infection induces augmentation of filamentous structures containing septin 2. These filaments co-localize with septin 9. HCV-infected Huh7.5 cells exhibit a well-developed microtubule network, which is less evident in non-infected cells. Septin 9 filaments co-localize with the microtubule network. siRNA silencing of septin 9 disintegrates the microtubule network. As a consequence, size and number of lipid droplets is increased in HCV-infected cells [62]. The lipid droplets and core protein co-localize in perinuclear regions. Knocking down septin 9 reduces size and number of LDs and HCV genomic RNA. Analysis of the transcript level of septin 9 variants showed that mRNA of septin 9 isoform 1 (septin 9_i1) is the most abundant. Thus, HCV may optimize the conditions for its replication by recruiting host septin 9 to modulate growth of lipid droplets and build up a lipid-enriched environment.

### 3.3. Influenza Virus/Newcastle Disease Virus

Highly-pathogenic avian influenza (HPAI) H5N1 and Newcastle disease (ND) viruses are causing substantial losses in poultry worldwide. Both can lead to central nervous system dysfunction including acute encephalitis in poultry and migratory birds. Balasubramaniam et al. [63] used mRNA differential display technique to analyze differentially expressed transcripts during HPAI H5N1 and NDV infection of chicken brains. The gene for septin 5, a vesicle and membrane associated protein involved in exocytosis, was among the upregulated genes after infection with HPAI H5N1; infection with NDV did not change the septin 5 expression levels. Thus, in different virus species, different mechanisms involving septins might be implicated in causing neurovirulence. The interaction of septin 5 and chicken brain proteins after infection with H5N1-avian influenza virus was also studied by Khairat et al. [64]. Expression of septin 5 in infected and uninfected brain samples was tested by immunohistochemistry. Septin 5 was detected in control and infected samples. The staining intensity was clearly stronger in infected tissues indicating an upregulation of septin 5 protein at the area of infection. Co-IP assays of brain lysates were performed to find proteins interacting with septin 5 during H5N1 infection. In addition to other host proteins, several septins were identified. While septin 7 was also present in control samples, septin 2, septin 6, and an isoform of septin 11 appeared exclusively in infected tissue underlining the differences in septin expression patterns caused by viral infections.

Increasing the multiplicity of infection (MOI) and infection time of H1N1 virus led to higher SEPT9_i1 mRNA expression levels in A549 cells. Downregulation of SEPT9_i1 mRNA reduced HIF-1α nuclear translocation in the infected cells. Viral replication was also inhibited evidenced by a decrease of viral M gene expression. It was shown that the JNK signaling pathway regulates the expression of SEPT9_i1 in A549 cells during H1N1 virus infection [65].

### 3.4. Human Herpesvirus 8 (Kaposi Sarcoma-Associated Herpesvirus)

Human herpesvirus 8 (HHV-8) is associated with Kaposi’s sarcoma and several other human malignancies. The latent HHV-8 protein kaposin A has oncogenic potential and is the most abundant protein in Kaposi’s sarcoma. Using phage display technique, a septin 4 variant was found to bind to kaposin A [66]. The septin 4 variant showed pro-apoptotic properties in transfected cells. It led to rounding up and detaching of cells. Caspase-3 activity was increased and the NF-ĸB signal transduction pathway was activated. Co-expression of the viral protein kaposin A had an antagonistic effect, decreasing the number of cells that were rounded up by diminishing caspase-3 activity and by inhibiting NF-ĸB signaling. These results suggest that expression of the viral kaposin A could interfere with the apoptotic properties of the septin 4 variant.

### 3.5. Flaviviruses

Zika virus (ZIKV) targets neural progenitor cells in the brain and induces congenital neurodevelopmental birth defects (e.g., microcephaly). Li et al. [67] hypothesized that ZIKV proteins might impact on the function of proteins involved in neuronal cell division during development. Importantly, the septin cytoskeleton is required for midbody formation during cytokinesis [68]. A fusion protein ZIKV NS2B3, consisting of the N-terminal NS3 protease domain and the NS2B protein, cleaves host septin 2, which leads to the disruption of septin 2 and septin 7 complexes. When these complexes are missing in the midbody during telophase, consequently cytokinesis is delayed with a failed mitotic abscission. Cytokinesis could be partially restored by forced expression of a non-cleavable septin 2. However, this study emphasizes on the importance of the function of septins in the pathogenesis of ZIKV infection.

Own research with Japanese encephalitis virus (JEV), another flavivirus, indicates that septin 7 may play a role in cellular infection and viral reproduction. In susceptible cells allowing complete virus propagation, infected cells tend to increase expression of septin 7 (unpublished data). However, it is not yet clear whether septin 7 is recruited around replicative viral RNA.

## 4. Summary and Conclusions

The 13 human septin genes together with alternative splice variants, tissue specific expression, and possible post-translational modifications create a complex cytoskeletal network with huge diversity. Such diversity might explain the multiplicity of subcellular localizations and the numerous functions in the cell metabolism [69]. Deregulation of septin expression levels therefore affects many cellular processes. In many cancers, septin expression levels are altered. In most of the cases, septins are over-expressed, but also down-regulation, ectopic expression of septins with unique tissue- and cell type-specific expression, and epigenetic alterations have been documented [70,71]. Septins can therefore be anti- or pro-oncogenic. Viral infections strongly alter the expression levels of a large number of genes [72,73,74] and members of the septin family are also found among those proteins. The link viral infection-septin is not straightforward: some septins have an anti-viral effect (septin 1/2/7/9/11), while others exhibit pro-viral properties (septin 3/10) [58,59]. Thus, deregulation of a certain septin can be induced by the infecting agent to establish optimal conditions for the infection cycle or represent a reaction of the host defense mechanism to fight the invading pathogen. Which septin (or septins) effectively is deregulated depends on the virus species [63] and the host cell type.

Septins may contribute to several steps of the virus replication cycle. Septin scaffolding platforms might be important for the recruitment of receptors to the cell surface during virus docking. Phagosomes equipped with particular septins possibly act as an entry mechanism for microorganisms or could be involved in intracellular transport. Invasion of pathogens triggers a range of cellular defense mechanisms. One strategy is the aggregation of septin filaments around infectious particles [54]. These structures may help with the elimination of the infectious agent by scaffolding the autophagy machinery around the pathogen [33].

However, more research is needed to fully understand the septin code [13] with its transcript diversity, the subcellular distribution of the various isoforms, the existence of different hexa- or octameric complexes, and the divers higher-order structures.

Development of small molecules disturbing the function or structure of septins may provide a possible target for anti-viral therapies. Due to the multiplicity of septin functions, direct targeting could have many side effects. Therefore, septin-specific and function-specific targeting would be needed. An alternative therapeutic approach might aim at the perturbation of the subcellular localization of septin filaments [69].
